# Comprehensive Characterization of *Lantana camara* Essential Oil from Angola: GC-MS Profiling, Antioxidant Capacity, and Drug-likeness Prediction

**DOI:** 10.3390/antiox15030291

**Published:** 2026-02-26

**Authors:** Nswadi Kinkela, Abdy Morales, Hugo A. Sánchez-Martínez, Maricselis Díaz, Nsevolo Samba, Monizi Mawunu, Juan A. Morán-Pinzón, Lúcia Silva, Jesus M. Rodilla, Estela Guerrero De León

**Affiliations:** 1 Chemistry Department, University of Beira Interior, 6201-001 Covilhã, Portugalmlas@ubi.pt (L.S.); rodilla@ubi.pt (J.M.R.); 2 Fiber Materials and Environmental Technologies (FibEnTech), University of Beira Interior, 6201-001 Covilhã, Portugal; 3 Department of Agronomy, University Kimpa Vita, Uige CP-77, Angola; 4 Centro de Investigaciones Psicofarmacológicas, Universidad de Panamá, Panama 3366, Panama; moba245@gmail.com (A.M.); juan.moran@up.ac.pa (J.A.M.-P.); 5 Departamento de Farmacología, Facultad de Medicina, Universidad de Panamá, Panama 3366, Panama

**Keywords:** *L. camara*, β-caryophyllene, α-humulene, antioxidant, essential oil

## Abstract

*Lantana camara* L. (*Verbenaceae*) is a medicinal plant widely used in traditional medicine in Angola, especially for its anti-inflammatory effects. This study evaluated the chemical composition of *L. camara* essential oil from leaves (Lc-EO) collected in Uíge Province, Angola. GC–MS analysis enabled the identification of 96 volatile compounds, with sesquiterpenes and monoterpenes as the predominant constituents. Among them, β-caryophyllene (14.49%), sabinene (9.13%), bicyclogermacrene (8.18%), α-humulene (5.66%), nerolidol (5.29%), and 1,8-cineole (5.14%) were identified as major components. The antioxidant activity of Lc-EO was assessed using DPPH, ABTS, and superoxide anion (O_2_^•−^) assays. Lc-EO showed strong activity in the DPPH assay (IC_50_ = 0.72 µg/mL), moderate activity in the ABTS assay (IC_50_ = 87.5 µg/mL), but minimal effect on O_2_^•−^ radicals (IC_50_ = 1491 µg/mL). It also significantly inhibited lipid peroxidation (IC_50_ = 236.2 µg/mL). The anti-inflammatory activity of Lc-EO was assessed through its ability to inhibit protein denaturation, exhibiting a moderate effect with 28% inhibition. In silico ADMET predictions suggested drug-like properties and low predicted systemic toxicity for major compounds. The *Artemia salina* lethality assay indicated moderate general toxicity (IC_50_ = 154.1 µg/mL), whereas the MTT viability assay revealed higher cytotoxic potency of Lc-EO (IC_50_ = 31.58 µg/mL), highlighting model-dependent differences in sensitivity. Overall, *L. camara* essential oil shows relevant bioactivity consistent with its traditional use, particularly antioxidant and anti-inflammatory effects, while its cytotoxicity highlights the need for safety evaluation. These findings indicate that the assayed oil is a promising source of bioactive compounds, but further studies are required to support its development as a safe pharmaceutical raw material.

## 1. Introduction

*L. camara* L. is an invasive plant species widely distributed across multiple regions worldwide [1]. In Angola, it is known by vernacular names such as “Cambumbulu,” “Cambumbe,” and “Flor-de-cerca,” particularly in areas where Kikongo, Kimbundu, and Umbundu are spoken. Although often regarded as a weed, the species is traditionally used in folk medicine primarily for the management of inflammatory conditions and topical skin applications [2,3], uses that are consistent with the biological activities investigated in the present study. In addition to its medicinal uses, *L. camara* has attracted scientific interest for its pesticidal, antimicrobial, and larvicidal activities, which have been validated in several studies [4,5,6].

Phytochemical analyses of *L. camara* essential oil, especially from leaves, have consistently revealed a predominance of mono- and sesquiterpenes, including β-caryophyllene, α-humulene, bicyclogermacrene, and related constituents [7,8,9]. These terpenoid compounds have been associated with antioxidant and anti-inflammatory properties [10,11,12,13], while their lipophilic nature may also contribute to cytotoxic or irritant effects, underscoring the importance of concurrent safety evaluation. However, previous studies have shown that different varieties of *L. camara*, influenced by geographical origin, plant parts, and environmental conditions, may contain varying types and concentrations of lantadenes and other phytochemicals, resulting in distinct bioactive profiles [14,15,16].

Despite the widespread traditional use of *L. camara*, information regarding the chemical variability, antioxidant activity, and safety profile of its essential oil from Angola remains limited. We hypothesized that essential oil obtained from leaves collected in Uíge Province exhibits chemical compositions associated with measurable antioxidant activity and model-dependent cytotoxic effects. Therefore, this study aimed to (i) characterize the chemical composition of leaf essential oil, (ii) evaluate its antioxidant and anti-inflammatory activities, and (iii) assess preliminary safety through in silico ADMET predictions and in vitro cytotoxicity assays. This approach allows a balanced evaluation of both bioactivity and safety within the analytical scope of the study.

## 2. Materials and Methods

### 2.1. Location and Origin of Samples

**Figure 1:**
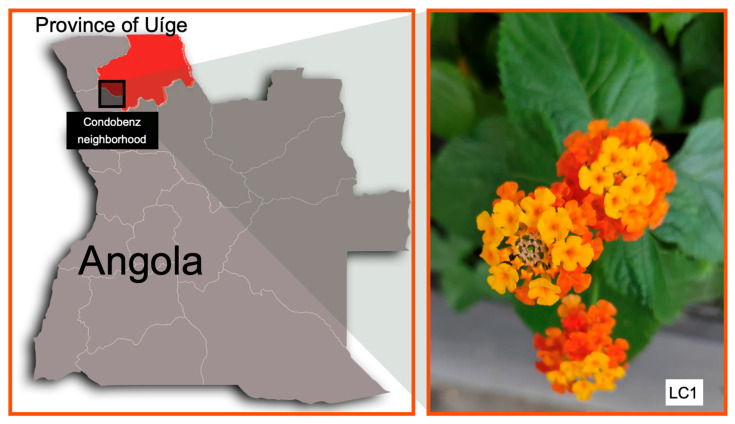
Geographic location of sample collection of *L. camara*.

Leaves of *L. camara* L. were collected in November 2022 from three individual plants located in the Condobenz neighborhood, Uíge Province, northern Angola (7°35′59″ S, 15°00′13″ E) (Figure 1). After collection, the leaves were carefully homogenized to form a composite sample representative of the local population. Taxonomic identification was performed by Professor Mawunu Monizi, Department of Agronomy, Kimpavita University (Uíge, Angola).

### 2.2. Preparation of Essential Oil

Fresh *L. camara* leaves were air-dried in a shaded environment protected from direct sunlight for 5 days to preserve the integrity of the active compounds and the natural coloration. After drying, the material was stored in plastic bags until distillation.

The essential oil was obtained by hydrodistillation using a Clevenger-type apparatus, with a solid-to-liquid ratio of 1:8 (g/mL). Distillation was carried out in triplicate, each run lasting three hours. At the end of each distillation, the essential oil was carefully separated from the aqueous phase, dried over anhydrous sodium sulfate, and filtered to remove residual moisture. Then the essential oil from *L. camara* leaves (Lc-EO) was stored in amber glass vials with airtight seals and kept under refrigeration (4 °C) until analysis.

Since the three distillations were performed using the same plant material and yielded similar results, only one representative oil sample was used for subsequent chemical and biological analyses. The yield was calculated based on the dry weight of the plant material and the volume of oil obtained.

### 2.3. Gas Chromatography-Mass Spectrometry Analysis

The chemical composition of Lc-EO was analyzed using Gas Chromatography coupled to Mass Spectrometry (GC-MS), a technique that enables efficient separation and precise identification of volatile and semi-volatile compounds.

GC-MS analyses were performed using an Agilent 7890A gas chromatograph equipped with a DB-5 capillary column (30 m × 0.25 mm, film thickness: 0.25 µm, J&W Scientific, Folsom, CA, USA) and coupled to an Agilent 5975C Inert XL MSD mass spectrometer with a triple-axis quadrupole detector. A 1 µL aliquot of each essential oil sample was injected in splitless mode at an injector temperature of 250 °C. The oven temperature program started at 50 °C (held for 2 min), followed by a temperature ramp of 3 °C/min to 190 °C and 10 °C/min to 300 °C, for each sample, to ensure optimal separation of the components. Helium was used as the carrier gas at a constant flow rate of 1 mL/min.

Mass spectra were acquired using electron impact (EI) ionization at 70 eV, with the ion source temperature set at 230 °C. Data acquisition and processing were carried out using ChemStation software C.01.10 (Agilent Technologies). The identification of the compounds was performed based on the Kováts Index (KI), which considers the chromatographic behavior of the substances. This index was used as a tool to characterize and differentiate the compounds present in essential oils, ensuring accurate identification. Compound identification was performed by comparing the obtained mass spectra with reference spectra from the NIST Mass Spectral Library (NIST/EPA/NIH).

### 2.4. In Silico Prediction of ADME and Toxicity Properties

The absorption, distribution, metabolism, and excretion (ADME) properties of the identified compounds were evaluated using the OSIRIS Property Explorer (https://www.organic-chemistry.org/prog/) (accessed on 12 May 2025) and the SwissADME platform (http://www.swissadme.ch) (accessed on 2 May 2025) [17]. OSIRIS was employed to predict key physicochemical and drug-likeness parameters, including calculated lipophilicity (cLogP), aqueous solubility (cLogS), molecular weight (MW), topological polar surface area (TPSA), drug-likeness, and drug score. Gastrointestinal absorption (GIA) and blood–brain barrier (BBB) permeability were predicted using the BOILED-Egg model available on the SwissADME platform [18].

Assessment of oral bioavailability was conducted according to Lipinski’s Rule of Five (Ro5) [19], which considers a compound drug-like if it fulfills the following criteria: molecular weight < 500 Da, logP ≤ 5, hydrogen-bond donors (HBD) ≤ 5, hydrogen-bond acceptors (HBA) ≤ 10, and no more than one violation of these rules.

Toxicity risk prediction was performed using two independent web-based platforms: PASS (http://www.way2drug.com/passonline) (accessed on 19 May 2025) and ADMETlab 3.0 (https://admetlab3.scbdd.com) (accessed on 13 May 2025) [20]. A compound was considered to have a potential toxicological risk if the predicted outcome was classified as “active” or if the model’s confidence score was ≥0.7 (ADMETlab 3.0). Additional predictive platforms were employed for comparative purposes, and their extended outputs are provided in the Supplementary Materials.

### 2.5. Evaluation of Antioxidant Activity of Lc-EO

The Lc-EO was dissolved in DMSO and vortex-mixed at 2000 rpm for 2 min to ensure homogeneous dispersion prior to the in vitro assays.

#### 2.5.1. DPPH Radical Scavenging Assay

The percentage inhibition of the DPPH (2,2-diphenyl-1-picrylhydrazyl), ABTS (2,2′-azino-bis(3-ethylbenzothiazoline-6-sulfonic acid)) radical was determined following the methodology proposed by Jianu et al. [21]. In each well of a 96-well microplate, 100 µL of DPPH solution and 100 µL of Lc-EO, previously dissolved in DMSO, were added at concentrations ranging from 15.6 to 500 µg/mL. Quercetin was used as the positive control. A negative control consisting of DMSO at concentrations equivalent to those used in the test samples was included and showed no antioxidant activity under the experimental conditions. The microplate was incubated in the dark at room temperature for 30 min, and the absorbance was measured at 492 nm using an EPOCH™).

All assays were performed in triplicate. The DPPH radical scavenging activity was expressed as the percentage of inhibition, calculated using Formula (1).

(1) $$Free\;radical\;scavenging\;\left(\%\right)=\left[A_{control}-A_{test}\right]\times 100/A_{control}$$

where A_control_ is the absorbance of the reaction media without the test sample, and A_test_ is the absorbance in the presence of the essential oil or Quercetin.

#### 2.5.2. ABTS Radical Cation Scavenging Assay

The free radical scavenging capacity of Lc-EO was evaluated as described by Valarezo et al. [22]. Two stock solutions were prepared: ABTS (2,2′-Azino-bis(3-ethylbenzthiazoline-6-sulfonic Acid)) (7.4 µM) and potassium persulfate (2.6 µM). Equal volumes of both solutions were mixed under constant stirring and incubated in the dark for 12 h to generate the ABTS working solution. Subsequently, 333 µL of the resulting solution was diluted with 20 mL of methanol to adjust the absorbance to 1.1 ± 0.02 at 734 nm, as measured using a UV–Vis spectrophotometer (EPOCH™ microplate reader, model M491, BioTek Instruments, Inc., Winooski, VT, USA).

For the assay, 10 µL of Lc-EO was mixed with 190 µL of the ABTS working solution in a 96-well plate and tested at various concentrations (15.6–500 µg/mL). The mixture was incubated at room temperature for 2 h, after which the absorbance was recorded at 734 nm. Quercetin was used as the positive control, and DMSO at a maximum concentration of 5% served as the negative control. The ABTS radical scavenging activity (%) was calculated using Formula (1).

#### 2.5.3. NBT Superoxide Radical Scavenging Assay

The superoxide anion radical (O_2_^•−^) scavenging activity was evaluated using a non-enzymatic system, following the method described by Saha et al. [23]. The assay was conducted in 96-well microplates. A volume of 50 μL of the Lc-EO, prepared to assay various concentrations (15.6 to 500 µg/mL), was mixed with 50 µL of each of the following reagents: phenazine methosulfate (PMS, 120 µM), nicotinamide adenine dinucleotide (NADH, 936 µM), and nitroblue tetrazolium (NBT, 300 µM). The reaction mixtures were incubated at 25 °C for 5 min, after which the absorbance was measured at 560 nm using a microplate reader as previously described.

All experiments were performed in triplicate for each concentration tested. The percentage of superoxide scavenging activity was calculated using the same formula applied for the DPPH radical inhibition assay.

#### 2.5.4. Lipid Peroxidation Inhibition Assay

The effect of Lc-EO (15.6 to 500 µg/mL) on egg yolk lipid peroxidation was assessed using the method described by Ruberto et al. [24], which quantifies malondialdehyde (MDA), a marker of fatty acid peroxidation. This assay was selected as a preliminary screening model for lipid peroxidation inhibition, acknowledging its simplified nature and limited extrapolation to in vivo systems. Briefly, 100 μL of egg yolk homogenate (1:25 *v*/*v* in phosphate-buffered saline, PBS, pH 7.4) was mixed with 10 µL of the essential oil, 50 μL of FeSO_4_ (25 mmol/L), and 300 µL of PBS. The mixture was incubated at 37 °C for 15 min, after which 50 µL of 15% (*w*/*v*) trichloroacetic acid (TCA) was added to stop the reaction. The samples were centrifuged at 3500 rpm for 15 min, and the resulting supernatants were collected. The absorbance of the sample (15.6 to 500 µg/mL) was measured at 532 nm to quantify the MDA levels.

The percentage inhibition of lipid peroxidation was calculated using Formula (2).

(2) $$Inhibition\;of\;lipoperoxidantion\;\left(\%\right)=\left[A_{control}-A_{test}\right]\times 100/A_{control}$$

where A_control_ is the absorbance of an egg yolk emulsion in a blank buffer without the test sample, and A_test_ is the absorbance of the egg yolk emulsion containing either the Lc-EO or the standard substance (quercetin).

### 2.6. Anti-Inflammatory Activity by Inhibition of Protein Denaturation

The in vitro anti-inflammatory activity of Lc-EO was evaluated using a method adapted from Gîlcescu Florescu et al. [25], optimized for use in 96-well microplates. In this assay, 5 µL of Lc-EO (at concentrations ranging from 15.6 to 500 µg/mL) was mixed with 245 µL of bovine serum albumin (0.4% BSA) prepared in phosphate-buffered saline (PBS, pH 6.4). The mixtures were incubated at 37 °C for 10 min, followed by heating at 70 °C for 5 min to induce protein denaturation. After cooling, the absorbance was measured at 660 nm using a microplate reader as previously described. Diclofenac was used as a positive control. A negative control was prepared under identical conditions, replacing Lc-EO with a solution of DMSO in proportions equivalent to those used in the test samples.

The percentage inhibition of albumin denaturation, which reflects the anti-inflammatory potential of the Lc-EO, was calculated using Formula (3).

(3) $$Inhibition\;of\;protein\;denaturation\;\left(\%\right)=\left[A_{control}-A_{test}\right]\times 100/A_{control}$$

where A_control_ is the absorbance of the negative control, and A_test_ is the absorbance of the sample containing either Lc-EO or diclofenac.

### 2.7. Toxicity Test of Lc-EO

#### 2.7.1. Toxicity Test with *Artemia salina*

The toxicity of Lc-EO was evaluated using the *Artemia salina* (EG Artemia, SEP-Art^®^, Great Salt Lake, UT, USA) bioassay, adapted for 96-well microplates as described by Mesquita et al. [26]. In each well, 100 µL of seawater containing 10 to 15 *A. salina* larvae was combined with 98 µL of artificial seawater (ASW) Instant Ocean (United Pet Group, Blacksburg, VA, USA) (prepared by dissolving 35 g/L in distilled water) and 2 µL of Lc-EO or DMSO (control), to achieve final concentrations ranging from 15.5 to 300 µg/mL. After 24 h of exposure, toxicity was assessed by determining the percentage of dead larvae in each well.

The median lethal dose (LD_50_) was defined as the concentration required to cause 50% mortality of Artemia salina nauplii. Based on established criteria, samples with LC_50_ values greater than 1000 µg/mL were considered non-toxic, whereas those with LD_50_ values between 500 and 1000 µg/mL were classified as weakly toxic, values between 100 and 500 µg/mL as moderately toxic, and values below 100 µg/mL as strongly toxic, according to Meyer et al. (1982) and Nguta et al. (2012) [27,28].

#### 2.7.2. MTT-Based Cytotoxicity Screening in RAW 264.7 Macrophages

RAW 264.7 murine macrophages were obtained from the National Centre for Cell Science (NCCS, Pune, India) and cultured in high-glucose DMEM as described by Taciak et al. [29]. Cytotoxicity was evaluated using the MTT (3-[4,5-dimethylthiazol-2-yl]-2,5 diphenyl tetrazolium bromide) assay, which quantifies mitochondrial dehydrogenase activity via the reduction of MTT to formazan. Cells were seeded in 96-well plates (1.2 × 10^5^ cells/mL) and incubated for 24 h to allow adherence and proliferation, following protocols by Selvaraj et al. [30] and Marques et al. [31]. Subsequently, cells were treated with Lc-EO (12.5–300 µg/mL in DMSO) for 24 h at 37 °C and 5% CO_2_. In all experiments, the final DMSO concentration was kept below 0.5%, and DMSO at equivalent proportions was used as the negative control. After treatment, MTT solution (0.5 mg/mL) was added and incubated for 12 h. Formazan crystals were solubilized in acidified isopropanol, and absorbance was measured at 570 nm using a microplate reader (EPOCH, BioTek Instruments, Inc., USA). Cytotoxicity was expressed relative to untreated controls.

### 2.8. In Silico Prediction of Biological Activity

To predict the potential pharmacological effects and molecular targets associated with the compounds identified in the Lc-EO, the PASS (Prediction of Activity Spectra for Substances) online platform was employed (http://www.way2drug.com/passonline) (accessed on 19 May 2025) [32].

For each compound, predicted activities are expressed as probability values: Pa (probability to be active) and Pi (probability to be inactive). In this study, only those predicted activities with Pa ≥ 0.7 were considered significant and retained for further analysis, as this threshold indicates a high likelihood of biological relevance.

### 2.9. Statistical Analysis

Data are expressed as mean ± standard deviation (SD). Statistical analyses were performed using GraphPad Prism software (version 10.0; GraphPad Software Inc., San Diego, CA, USA). Differences among groups were assessed by one-way analysis of variance (ANOVA), followed by Tukey’s multiple comparisons test. Dose-response curves were fitted by nonlinear regression using a four-parameter logistic model with variable slope. In the anti-inflammatory activity assay, IC_50_ values were estimated relative to the fitted Emax due to the limited maximal efficacy of Lc-EO (~72%), rather than by direct interpolation. Statistical significance was denoted as * *p* < 0.05, ** *p* < 0.01, and *** *p* < 0.001.

## 3. Results

### 3.1. Yields and Phytochemical Characterization of Essential Oil

The essential oil yields from leaf samples of *L. camara* were comparable, exhibiting minor variations in volume and yield (Table 1).

### 3.2. Phytochemical Characterization of Essential Oil

GC–MS analysis allowed the identification of 96 compounds in the Lc-EO, excluding non-identified peaks. The composition was dominated by sesquiterpenes (51.5%), followed by monoterpenes (27.3%), with minor proportions of aromatic compounds and other trace constituents.

**Figure 2:**
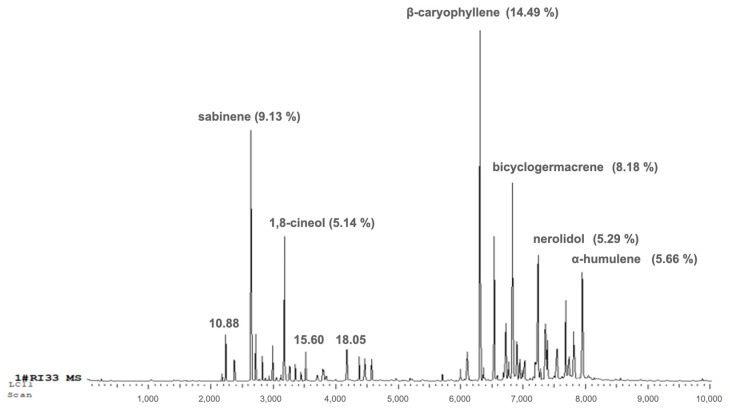
Representative GC-MS total ion chromatograms of Lc-EO collected from the Condobenz neighborhood, Angola.

The chromatogram (Figure 2) showed well-defined and high-intensity peaks corresponding to the major constituents. Among them, β-caryophyllene (14.49%), sabinene (9.13%), bicyclogermacrene (8.18%), α-humulene (5.66%), nerolidol (5.29%), and 1,8-cineole (5.14%) were the most abundant compounds, together accounting for 47.89% of the total oil composition.

In addition to the major constituents, other characteristic components were identified in the Lc-EO sample, including α-pinene, β-pinene, δ-3-carene, 2-bornanone, 1-octen-3-ol, and Davana ether 1. Table 2 presents the complete list of volatile constituents identified in the analyzed essential oil.

### 3.3. Prediction of ADME and Toxicity Properties

Computational ADMET analysis indicated that major compounds possess drug-like properties, with only one violation of Lipinski’s Rule of Five (Table 3). The compounds displayed a range of lipophilicity (cLogP 2.11–6.24), with α-humulene being the most lipophilic (6.24) and 1,8-cineole the least (2.11), while all showed low water solubility (cLogS −2.48 to −3.66). 1,8-cineole (eucalyptol) and nerolidol demonstrated high gastrointestinal absorption (GIA), and three compounds (sabinene, 1,8-cineole, and nerolidol) were predicted to cross blood–brain barrier (BBB). All compounds were predicted not to be substrates of P-glycoprotein (P-gp), suggesting a lower likelihood of active efflux, which may enhance their intracellular bioavailability.

On the other hand, the major compounds of Lc-EO showed no predicted structural alerts for pan-assay interference (PAINS) and yielded synthetic accessibility scores ranging from 2.87 to 4.51. However, sabinene exhibited the highest predicted drug score (0.45).

Toxicological prediction identified six compounds with potential irritant effects, with skin sensitization, carcinogenicity, respiratory effect, and hepatotoxicity being the most frequently predicted toxicological endpoints.

Despite the utility of tools for ADMET profiling, these results must be interpreted as theoretical predictions requiring experimental validation. The predictive values are inherently constrained by the underlying algorithms and the varying quality of training data, which may suffer from class imbalance or subjective annotations.

### 3.4. Antioxidant Activity of Lc-EO

The antioxidant capacity of Lc-EO was evaluated using DPPH, ABTS, O_2_^•−^, and lipid peroxidation inhibition assays. The results are expressed as half-maximal inhibitory concentrations (IC_50_, µg/mL) and compared with the reference antioxidant quercetin (Table 4). 

The Lc-EO exhibited a strong radical-scavenging effect against DPPH with an IC_50_ value of 0.72 ± 0.2 µg/mL, markedly lower than that of quercetin (17.32 ± 3.2 µg/mL). This finding highlights a high efficiency of the oil in neutralizing DPPH radicals. Similarly, in the ABTS assay, the essential oil showed an IC_50_ of 87.5 ± 4.6 µg/mL, which, although higher than that of quercetin (15.8 µg/mL), still demonstrates relevant antioxidant potential.

In contrast, Lc-EO displayed limited scavenging activity against O_2_^•−^, with a high IC_50_ of 1491 µg/mL, indicating weak activity compared to quercetin (13.92 ± 9.1 µg/mL).

The lipid peroxidation assay further corroborated the antioxidant potential of Lc-EO, in agreement with the results obtained in the DPPH and ABTS radical scavenging assays. The Lc-EO sample achieved a maximum inhibition of 72.2 ± 1.4%, which, although lower than that observed for the reference compound quercetin (92.4 ± 0.1%), confirms its relevant antioxidant activity.

### 3.5. Anti-Inflammatory Activity of L. camara Essential Oil

**Figure 3:**
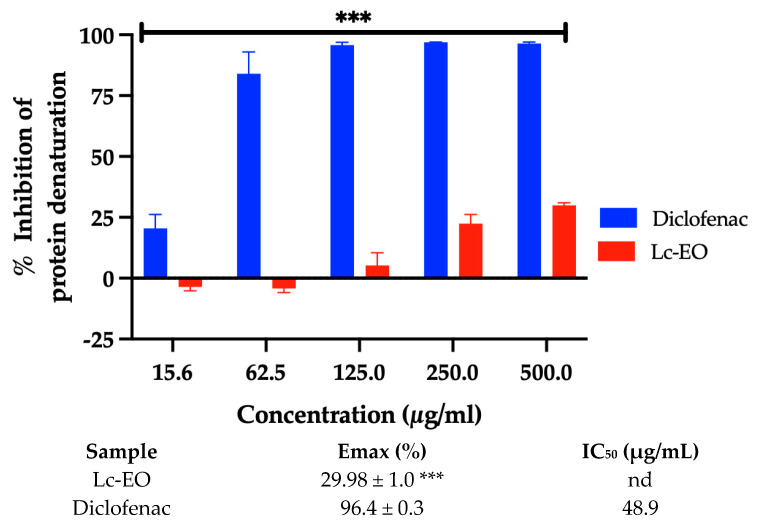
Percent inhibition (Emax) and half-maximal inhibitory concentration (IC_50_) of Lc-EO against protein denaturation assay. Emax data are presented as mean SD for n = 3. *** *p* < 0.001 vs. Diclofenac and. nd = not determined.

The anti-inflammatory activity of Lc-EO was evaluated based on its ability to inhibit protein denaturation (in vitro *model*). As shown in Figure 3, Lc-EO exhibited a moderate inhibitory effect compared to the reference drug diclofenac sodium (*p* < 0.05). While diclofenac achieved nearly complete inhibition (~100%) of protein denaturation, Lc-EO showed a dose-dependent effect, reaching a maximum inhibition of approximately 28% at the highest tested concentration. The IC_50_ value for Lc-EO could not be determined, as the maximum inhibition did not reach 50%, preventing reliable curve fitting. This was reported as “not determined” (nd), indicating limited anti-inflammatory activity within the tested concentration range.

### 3.6. Toxicity Assays

#### 3.6.1. Preliminary Toxicity Assessment Using the Artemia Salina

**Figure 4:**
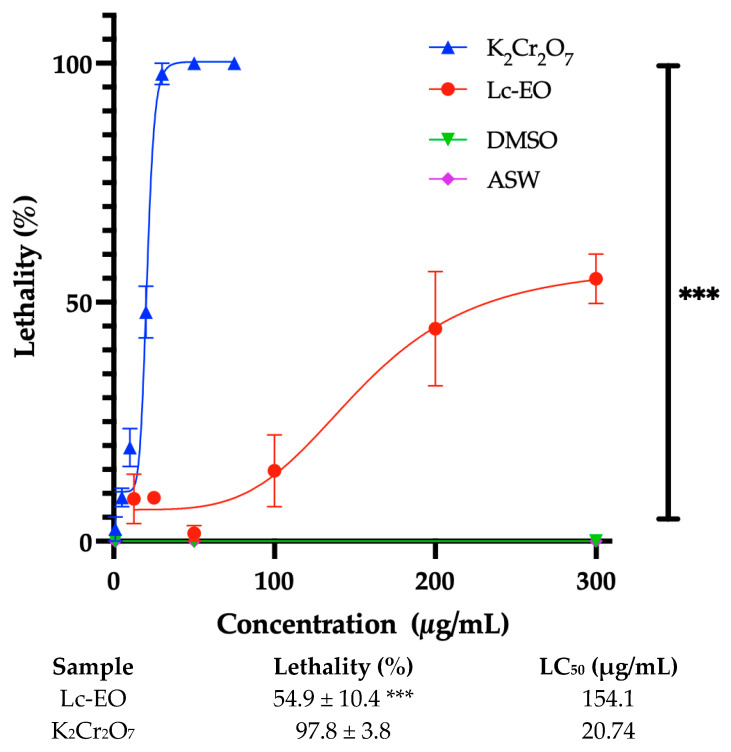
Maximum mortality data and lethal concentration 50 (LC_50_) obtained from the evaluation of Lc-EO in the toxicity model in *Artemia salina*. Lethality percentages are presented as the mean ± SD for n = 3. *** *p* < 0.001 vs. K_2_Cr_2_O_7_. ASW: artificial seawater.

No mortality was observed in the control group, indicating that 1% DMSO does not significantly affect nauplius viability (Figure 4). Lc-EO exhibited a concentration-dependent mortality, ranging from 10% to 60% across the tested concentrations. The Lc-EO induced a maximum mortality rate of 54.9 ± 10.4%, whereas the positive control (potassium dichromate) caused 97.8 ± 3.8% mortality. The calculated LC_50_ values were 154.1 µg/mL for the essential oil and 20.74 µg/mL for the standard.

#### 3.6.2. Cytotoxicity Screening in RAW 264.7

**Figure 5:**
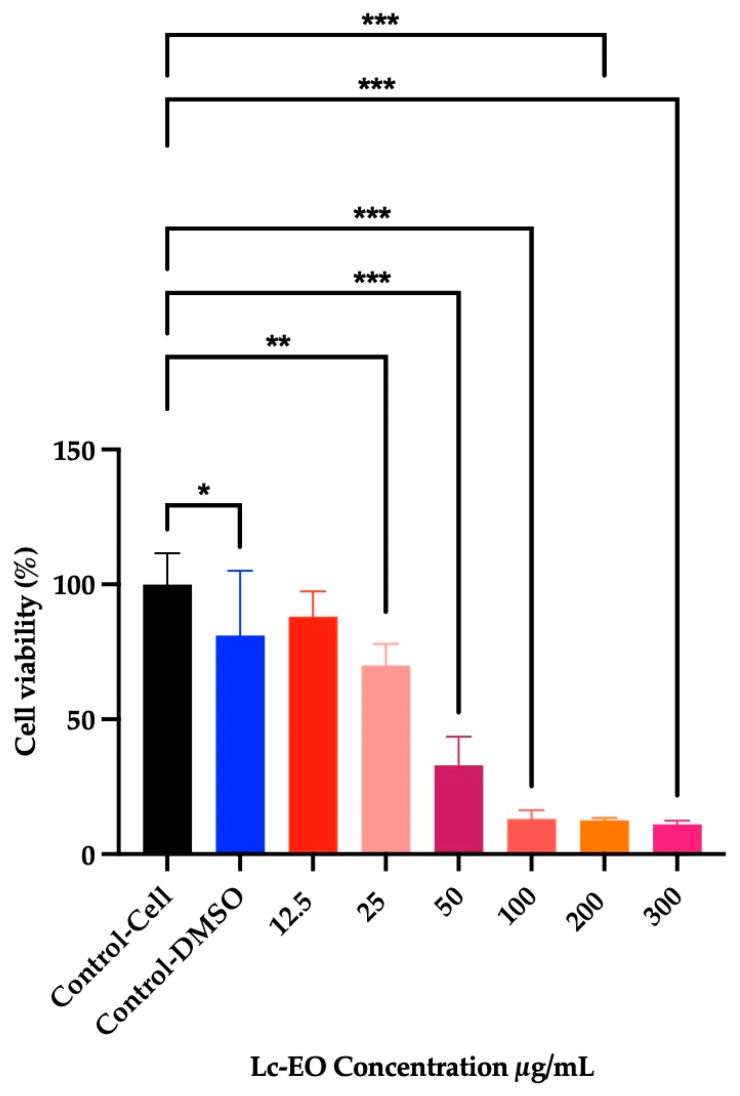
Cell viability as measured by MTT assay by treating cells with different concentrations of Lc-EO (12.5–300 μg/mL). Viability percentages are presented as the mean ± SD for n = 6. * *p* < 0.05, ** *p* < 0.01, and *** *p* < 0.001 vs. Control-cell.

The relative metabolic activity of RAW 264.7 macrophages decreased in a concentration-dependent manner following exposure to increasing concentrations of Lc-EO at 12.5, 25, 50, 100, 200, and 300 µg/mL (Figure 5). After 24 h of treatment, the IC_50_ value was determined to be 31.58 µg/mL, indicating a significant cytotoxic effect of Lc-EO. At the highest concentration tested (100 to 300 µg/mL), cell viability was reduced by up to 88% compared to untreated control cells.

### 3.7. Prediction of Biological Activity

Using the PASS online tool, we screened the six primary Lc-EO compounds for potential biological activities. Table 5 details the predicted activities for each compound. Our data indicate some interesting pharmacological activities, considering probably activity ≥ 0.7 as highly probable for experimental validation. The most highlighted evidence is targeted for anti-cancer, anti-inflammatory, and dermatologic applications. Some multiple-effect compounds, such as sabinene and β-caryophyllene, show broad activity (bone, skin, and cancer), and 1,8-cineole and nerolidol may benefit the liver, mental health, and some metabolic disorders.

## 4. Discussion

This study presents a comprehensive phytochemical, pharmacological, and toxicological evaluation of essential oil obtained from *L. camara* leaves collected in Uíge, Angola. The objective of determining the chemical composition of *L. camara* essential oil from Uíge Province, Angola, was motivated by previous reports indicating that chemical composition and relative abundance can vary according to geographical origin, climatic conditions, and soil characteristics [35]. Thus, the data from our study, consistent with previous reports, identify *L. camara* as a significant source of monoterpenes and sesquiterpenes, with β-caryophyllene, sabinene, and α-humulene as the main and most abundant compounds [5,6,36].

Several studies have shown that the chemical composition of *L. camara* exhibits high geographic variability, giving rise to different chemotypes. Satyal et al. (2016) [35] reported marked differences among samples from Yemen, Cuba, and Nepal, with significant changes in the proportions of monoterpenes and sesquiterpenes. Similarly, research conducted in Brazil identified distinct chemical profiles among populations of the species, highlighting relevant variations in the predominance of γ-curcumenes, zingiberene, and germacrenes [7,12]. In contrast, the essential oil from Uíge presents a sesquiterpene-rich chemotype characterized by high concentrations of β-caryophyllene and bicyclogermacrene, in addition to sabinene levels higher than those generally reported in the literature. This differentiation confirms the strong influence of environmental, edaphoclimatic, and genetic factors on the species’ ability to alter its chemical composition according to the surrounding conditions [6,26], indicating that the Uíge chemotype constitutes a unique phytochemical signature and reinforcing the importance of characterizing *L. camara* populations from different geographic regions.

The PASS online platform predicted various pharmacological activities for the major compounds identified in the Lc-EO sample. The most prominent predicted effects included antineoplastic, anti-inflammatory, and dermatological applications. Additional potential benefits included lipid metabolism regulation, antiulcer activity, and hepatoprotective effects. These computational predictions are consistent with findings from biological studies. For instance, anti-inflammatory activity has been previously described for α-humulene [10,37], sabinene [38], β-caryophyllene [39], 1,8-cineole [40], and nerolidol [41]. Nevertheless, these predicted activities should be regarded as exploratory indicators of potential biological relevance rather than confirmed therapeutic effects.

These compounds have also been reported to exhibit antioxidant properties [42,43,44], which are relevant to their proposed antitumor and anti-inflammatory activities. The findings regarding the individual pharmacological potential of *L. camara* constituents are further supported by this study, in which the Lc-EO demonstrated significant antioxidant activity. This activity was demonstrated by its strong capacity to scavenge the DPPH radical. Comparable results have been reported by other authors, who confirmed the antioxidant potential of *L. camara* leaf essential oil against DPPH [45,46,47]. The antioxidant capacity exhibited by Lc-EO is likely attributable to its principal constituents, and a similar synergistic antiradical effect has been documented in essential oils from other geographical origins [6,36]. Furthermore, individual contributions from major compounds such as β-caryophyllene are well-supported in the literature, as this sesquiterpene has demonstrated the ability to inhibit lipid peroxidation in both in vitro and in vivo models [48,49,50].

Regarding experimental anti-inflammatory activity, the essential oil exhibited a moderate effect, inhibiting 28% of protein denaturation, which was lower than the inhibition observed for diclofenac (Emax ≈ 100%). The protein denaturation assay has been previously employed by Khairan et al. (2024) to evaluate the anti-inflammatory potential of an ethanolic extract of *L. camara*, where the authors reported a concentration-dependent increase in inhibition of protein denaturation [2]. The anti-inflammatory properties described for the terpenoids present in *L. camara* are part of the findings that reinforce the results obtained in our study [51]. Collectively, these results demonstrate the potential anti-inflammatory effect of *L. camara*, whose magnitude appears to depend on the extraction method employed.

In terms of toxicity, previous studies have demonstrated that *L. camara* essential oil exhibits notable acaricidal activity, primarily attributed to major constituents such as γ-curcumene and nerolidol, both of which were also identified as abundant components in Lc-EO [52]. Beyond nerolidol, other predominant constituents, including bicyclogermacrene and α-humulene, have been reported to exert cytotoxic effects [43,53].

In our study, Lc-EO displayed moderate toxicity in the *Artemia salina* assay (IC_50_ = 154.1 μg/mL), according to the classification proposed by Rajabi et al. (2015) [54] and marked cytotoxicity in the MTT assay (IC_50_ = 31.58 μg/mL), suggesting a relationship with the antiparasitic action described for *L. camara* essential oil [6].

The pronounced cytotoxic response observed in the MTT assay, together with the presence of bioactive sesquiterpenes, provides strong support for the toxicological profile of Lc-EO and is consistent with the antitumoral and acaricidal activities previously reported for this species [11,52]. Thus, while the oil displays promising pharmacological potential, its therapeutic margin should be carefully evaluated. In this context, the detected cytotoxicity suggests that its practical applications may be limited, particularly for oral formulations, and reinforces the need for dose optimization and route-of-administration considerations.

Finally, it is important to emphasize that the use of *L. camara* by communities in Uíge is largely empirical and rooted in tradition, without formal scientific support. Although the plant is widely used for medicinal purposes, the findings of this study show that the local chemotype contains compounds with toxic potential, indicating that indiscriminate use may pose health risks. In this context, the data presented here provide an essential scientific basis to guide safer practices and support educational initiatives aimed at the population, contributing to a more informed and responsible use of the species.

## 5. Conclusions

This study highlights the importance of integrated phytochemical and biological characterization of *L. camara* in the context of its ethnobotanical use in Angola. The identification of bioactive terpenoid constituents, together with the observed antioxidant activity, moderate anti-inflammatory effects, and model-dependent cytotoxicity, provides partial pharmacological support for its traditional medicinal applications. Importantly, the detection of cytotoxic effects also emphasizes the need for careful safety assessment, dose consideration, and route-of-administration evaluation, as these factors may limit certain applications, particularly oral use. Collectively, these findings contribute mechanistic and experimental evidence that helps to contextualize the ethnomedicinal use of *L. camara* and supports a more rational, evidence-based, and informed utilization of this species.

## Figures and Tables

**Table 1:** Extraction yield of essential oil from *L. camara* leaves.

Sample No.	Reference	Location	Sample Mass (g)	V_O.E_ (mL)	Average Yield (%)
1	Lc-EO	Condobenz/Uíge, Angola	100	0.9	0.816
2	Lc-EO	Condobenz/Uíge, Angola	100	0.8	
3	Lc-EO	Condobenz/Uíge, Angola	100	0.7	

**Table 2:** Volatile compounds identified by gas chromatography–mass spectrometry (GC–MS) in the leaf essential oil of *L. camara* L., showing Kovats index (KI), molecular ions (M^+^), and relative percentages.

Lc-EO					
N°	Compounds	KI Experimental	KI Theoretical	M^+^	%
1	2-ethylfuran	705	690	96	Traces
2	Ethyl propanoate	714	≈710–715	102	Traces
3	2-methylbutan-1-ol	744	≈720–725	102	Traces
4	Butyl acetate	812	≈810–815	116	Traces
5	2-hexenal	854	≈850–860	98	Traces
6	3-hexen-1-ol	857	855–860	100	Traces
7	1,3-dimethylbenzene (m-xylene)	877	≈880	106	Traces
8	2-methylbutan-1-yl acetate	977	≈860–867	103	Traces
9	1,4-dimethylbenzene (*p*-xylene)	925	≈926–928	106	Traces
10	α-thujene	931	≈926–928	136	0.24
11	α-pinene	939	≈930–936	136	1.57
12	camphene	953	≈950–955	136	0.72
13	sabinene	976	≈973–976	136	9.13
14	β-pinene	980	≈971–980	136	1.16
15	1-octen-3-ol	980	≈980–985	128	1.64
16	6-methyl-5-hepten-2-one	985	≈986–990	126	Traces
17	β-myrcene	991	≈991–995	136	0.82
18	3-octanol	993	≈1002–1008	130	0.11
19	α-phellandrene	1005	≈1004–1010	136	0.18
20	δ-3-carene	1011	≈1011–1013	136	1.16
21	α-terpinene	1018	≈1016–1020	136	0.13
22	p-cymene	1026	≈1023–1026	134	0.22
23	limonene	1031	≈1024–1030	136	0.82
24	1,8-cineole (eucalyptol)	1033	≈1026–1033	154	5.14
25	*cis*-β-ocimene	1040	≈1038–1040	136	0.482
26	*trans*-β-ocimene	1050	≈1044–1048	136	0.55
27	γ-terpinene	1059	≈1058–1060	136	0.28
28	*trans*-sabinene hydrate	1089	≈1086–1090	154	0.96
29	*cis*-sabinene hydrate	1069	≈1070–1075	154	Traces
30	β-terpinolene	1086	≈1085–1090	136	Traces
31	α-terpinolene	1088	≈1085–1090	136	0.21
32	*trans*-sabinene hydrate isomer	1089	≈1086–1090	154	0.41
33	linalool	1098	≈1099–1101	154	0.35
34	2-methylbutyl 2-methylbutanoate	1100	-	172	0.18
35	*trans*-ment-2-en-1-ol	1136	-	154	Traces
36	β-terpineol	1159	≈1155–1165	154	Traces
37	2-bornanone (camphor)	1141	≈1139	152	1.22
38	myrcenone	1145	≈1148	150	Traces
39	endo-borneol	1165	≈1158–1165	154	0.91
40	terpinen-4-ol	1177	≈1177–1179	154	0.76
41	α-terpineol	1189	≈1190–1195	154	0.76
42	verbenone	1204	≈1204	150	0.08
43	iso-pinocamphyl angelate	1285	-	236	Traces
44	β-cyclocitral	1218	≈1220–1230	152	0.02
45	bornyl formate	1223	≈1220–1225	182	0.02
46	*cis*-ocimenone	1226	≈1225–1235	150	0.08
47	Neral	1240	≈1235–1240	150	0.10
48	carvone	1242	≈1242–1245	150	0.02
49	geraniol	1255	≈1254–1258	154	0.06
50	geranial	1270	≈1265–1270	152	0.11
51	perillaldehyde	1269	≈1268–1272	150	0.06
52	thymol	1290	≈1289–1290	150	0.04
53	carvacrol	1298	≈1298–1299	150	0.00
54	bicycloelemene	1333	≈1330–1340	204	0.02
55	γ-elemene	1430	≈1430–1435	204	0.26
56	α-cubebene	1351	≈1350–1355	204	0.04
57	eugenol	1356	≈1356–1358	204	0.06
58	α-copaene	1376	≈1376–1378	204	0.39
59	geranyl acetate	1383	≈1385–1388	196	0.04
60	β-elemene	1391	≈1389–1392	204	Traces
61	β-cubebene	1390	≈1390–1395	204	Traces
62	*cis*-jasmone	1391	≈1395–1400	204	1.20
63	1,1,7,7a-tetramethyl-1a,2,6,7,7a,7-hexahydro-1H-cyclopropa[a]naphthalene	1334	≈1335–1345	164	0.09
64	*cis*-β-ocimene	1339	≈1038–1040	202	0.03
65	aromadendrene	1439	≈1435–1440	204	Traces
66	α-gurjunene	1409	≈1405–1410	204	Traces
67	β-caryophyllene	1419	≈1417–1421	204	14.49
68	β-copaene	1392	≈1390–1395	204	0.47
69	aromadendrene isomer	1439	≈1435–1445	204	Traces
70	α-humulene	1452	≈1450–1455	204	5.66
71	valerena-4,7(11)-diene	1417	≈1415–1425	204	Traces
72	alloaromadendrene	1461	≈1455–1460	204	0.24
73	γ-muurolene	1477	≈1475–1480	204	0.37
74	germacrene D	1480	≈1477–1480	204	2.21
75	γ-amorphene	1495	≈1488–1493	204	0.06
76	bicyclogermacrene	1494	≈1490–1495	204	8.18
77	N.I.			220	0.16
78	Davana ether 1	1385	≈1380–1390	234	2.01
79	Cubebol	1518	≈1508–1515	222	0.93
80	δ-cadinene	1524	≈1522–1525	204	0.41
81	Davana ether 2	1398	≈1395–1405	234	0.74
82	N.I.			204	Traces
83	N.I.			202	0.14
84	Elemol	1547	≈1547–1550	222	0.25
85	davanone	1564	≈1560–1570	236	0.71
86	Germacrene B	1559	≈1555–1560	204	Traces
87	Nerolidol	1564	≈1560–1565	222	5.29
88	Spathulenol	1575	≈1575–1580	220	2.48
89	viridiflorol	1590	≈1590–1592	222	0.13
90	N.I.			220	0.35
91	N.I.			220	1.84
92	mixture (α-santalol)	1687	≈1680–1690	220	Traces
93	germacrene D-4-ol	1574	≈1570–1580	220	0.26
94	τ-muurolol	1641	≈1640–1645	222	Traces
95	Caryophyllene oxide	1581	≈1580–1585	220	3.27
96	iso-spathulenol	1623	≈1622–1627	220	0.53
97	τ-cadinol	1640	≈1640–1650	220	1.27
98	toreyol	1645	≈1645–1650	220	0.37
99	Mixture, N.I.			220	1.84
100	Epoxy-humulene	1607	≈1605–1615	220	0.89
101	N.I.			234	0.31
102	N.I.			220	4.63
103	2-hydoxydavanone	1627	≈1625–1635	252	0.22
104	N.I.			222	0.26

KI: Kovats index measured and compared with literature values [33,34]. N.I.: Not identified.

**Table 3:** Calculated ADME properties and in silico toxicity risk predictions for the major compounds identified in Lc-EO.

	Compound	cLogP ^3^	cLogS ^3^	MW ^3^	TPSA (Å^2^) ^3^	GIA ^‡^	BBB ^‡^	P-gp ^‡^	Lipinski (N° Violations) ^‡^	PAINS ^‡^	BS ^‡^	SA ^‡^	Dl ^3^	DS ^3^	Toxicity Risks ^†^
1	α-humulene	6.24	−3.4	204	0	Low	No	No	Yes (1)	0	0.55	3.66	−4.72	0.28	A^1,2^, D^2^
2	sabinene	2.86	−2.69	136	0	Low	Yes	No	Yes (1)	0	0.55	2.87	−6.78	0.45	A^1,2^, B^2^, D^1,2^, E^2^
3	bicyclogermacrene	5.53	−3.49	204	0	Low	No	No	Yes (1)	0	0.55	4.34	−4.88	0.07	A^1,2^, D^1,2^
4	β-caryophyllene	5.49	−3.66	204	0	Low	No	No	Yes (1)	0	0.55	4.51	−6.48	0.31	A^1,2^, D^1,2^
5	1,8-cineole (Eucalyptol)	2.11	−2.48	154	9.23	High	Yes	No	Yes (1)	0	0.55	3.65	−3.21	0.17	A^2^, D^2^
6	nerolidol	5.4	−3.12	222	20.23	High	Yes	No	Yes (1)	0	0.55	3.53	−6.38	0.19	A^1,2^, C^1^, D^2^, E^2^

MW: molecular weight; TPSA: topological polar surface area; GIA: gastrointestinal absorption; BBB: blood–brain barrier permeant; P-gp: P-glycoprotein protein substrate; PAINS: pan-assay interference compounds (n° Alerts); BS: bioavailability score; SA: synthetic accessibility; Dl: drug-likeness; DS: drug-score. ^†^ Toxicity risks: Irritant (A), carcinogenicity (B), hepatotoxicity (C), skin sensitization (D), respiratory effects (E); data obtained by PASS ^1^, ADMETlab3.0 ^2^, Osiris ^3^. ^‡^ Obtained by SwissADME.

**Table 4:** Half-maximal inhibitory concentration (IC_50_) obtained with Lc-EO tested against the DPPH, ABTS^•^ and O_2_^•−^ radicals, and lipid peroxidation.

	DPPH	ABTS	O_2_^•−^	Lipid Peroxidation
Lc-EO	0.72 ± 0.2	87.51 ± 4.6	1491.00 ± 76.6	236.22 ± 34.4
Quercetin	17.32 ± 3.2	15.83 ± 1.1	13.92 ± 9.1	9.74 ± 4.5

**Table 5:** Pharmacological activities of Lc-EO majoritarian compounds: a computational assessment.

Compounds	High-Confidence Pharmacological Effects Predictions by PASS Online ^†^	Pa	Pi
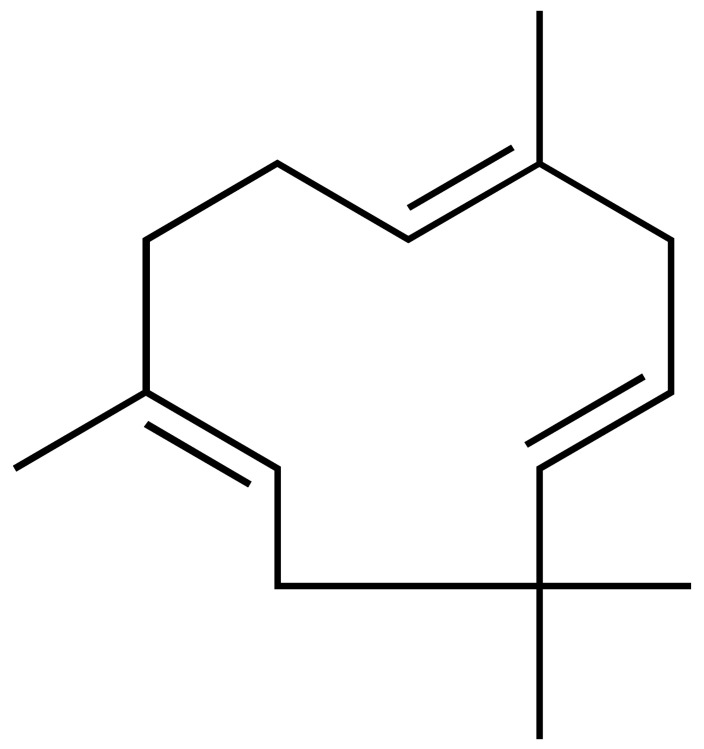 α-humulene	Antineoplastic	0.835	0.008
	Antieczematic	0.819	0.015
	Anti-inflammatory	0.741	0.011
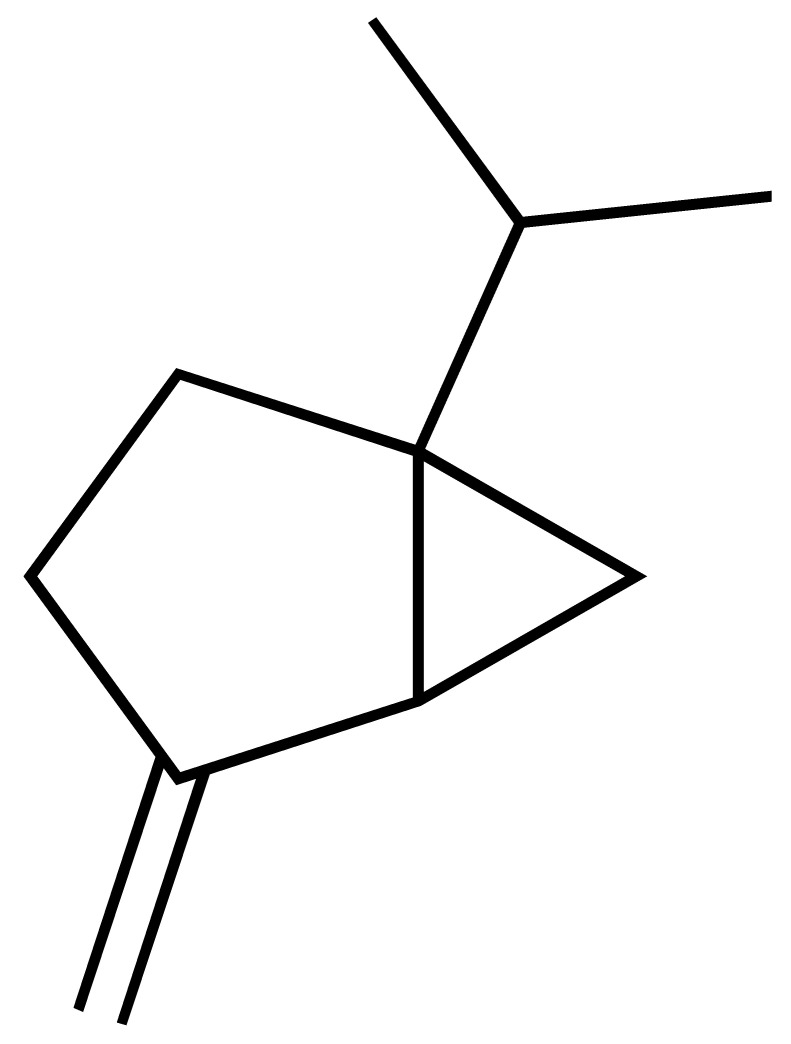 Sabinene	Antieczematic	0.947	0.003
	Antineoplastic	0.891	0.005
	Antiinflammatory	0.853	0.005
	Antipsoriatic	0.800	0.004
	Bone diseases treatment	0.782	0.005
	Dermatologic	0.757	0.005
	Antiosteoporotic	0.743	0.005
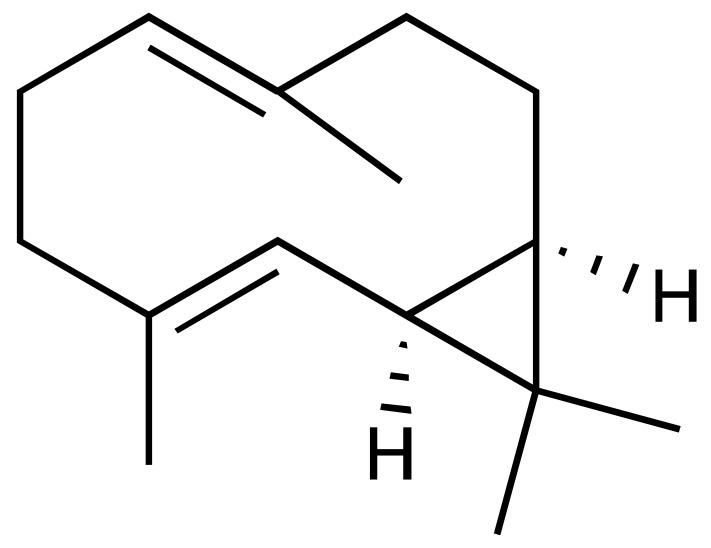 Bicyclogermacrene	Antieczematic	0.835	0.012
	Phobic disorders treatment	0.753	0.053
	Analgesic	0.7	0.010
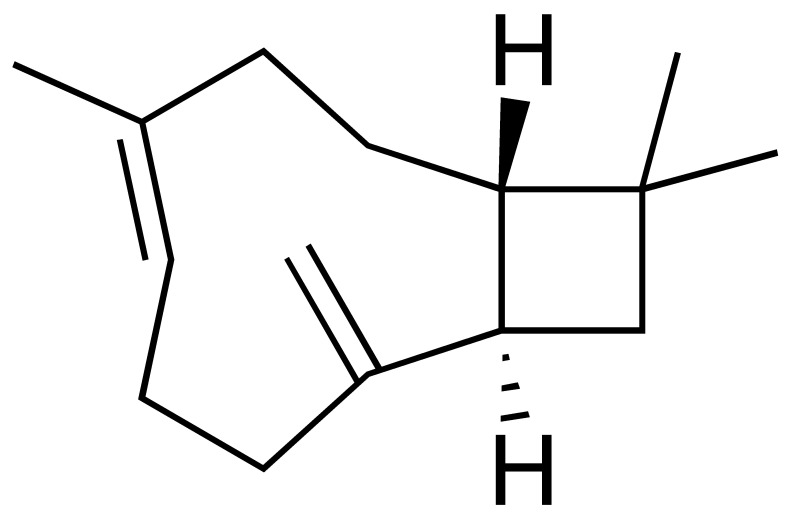 β-caryophyllene	Antineoplastic	0.915	0.005
	Antieczematic	0.897	0.005
	Antineoplastic (lung cancer)	0.763	0.005
	Antiinflammatory	0.745	0.011
	Antipsoriatic	0.734	0.005
	Dermatologic	0.734	0.006
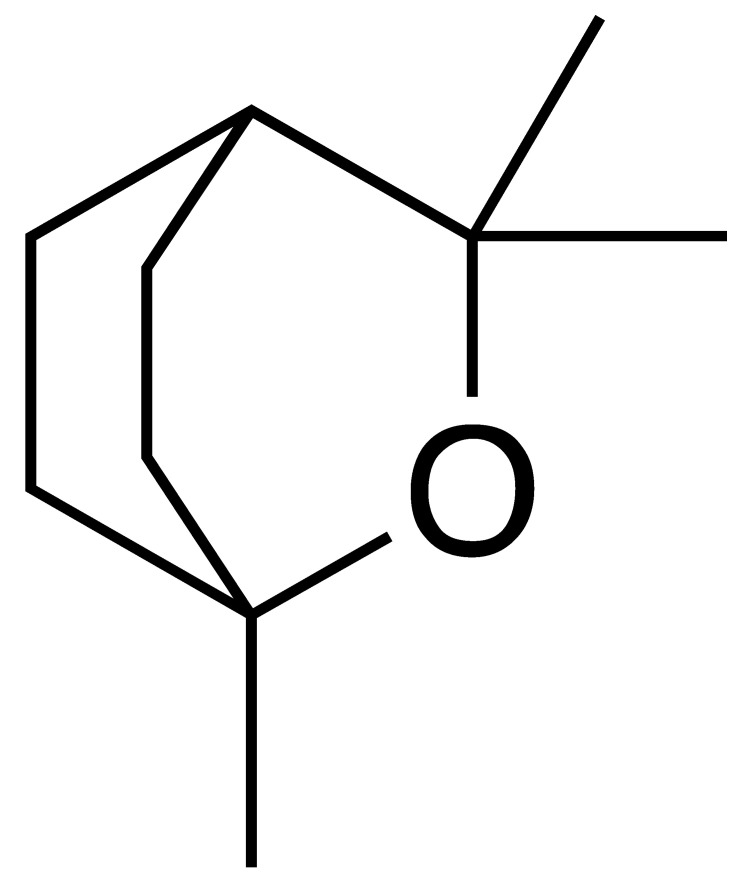 1,8-cineole	Phobic disorders treatment	0.833	0.022
	Hepatic disorders treatment	0.793	0.004
	Antineoplastic (lung cancer)	0.777	0.004
	Antidyskinetic	0.778	0.007
	Antineoplastic (colorectal cancer)	0.755	0.005
	Antineoplastic (colon cancer)	0.751	0.005
	Antiprotozoal	0.744	0.004
	Antiseborrheic	0.730	0.032
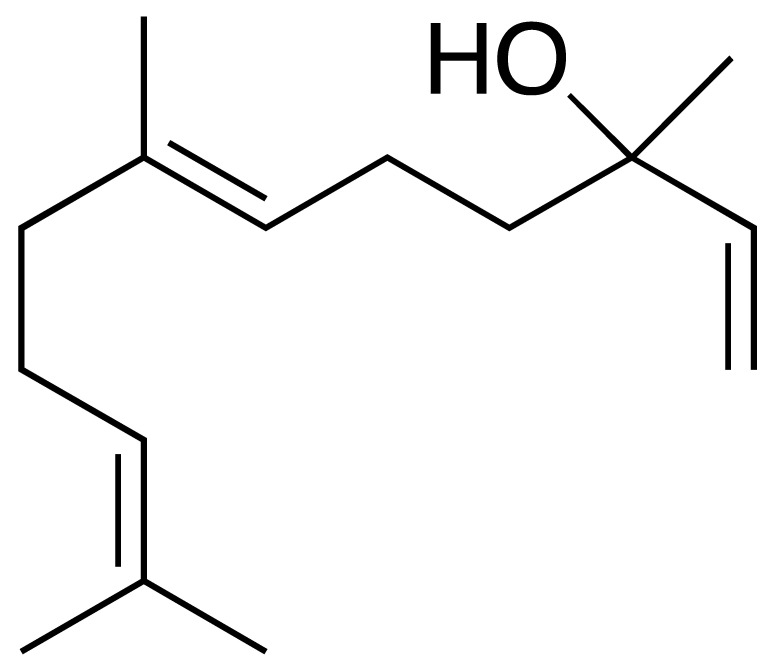 Nerolidol	Mucomembranous protector	0.983	0.002
	Lipid metabolism regulator	0.861	0.004
	Antisecretoric	0.843	0.004
	Anti-inflammatory	0.800	0.007
	Antihypercholesterolemic	0.781	0.005
	Antiviral (Rhinovirus)	0.765	0.001
	Antiulcerative	0.763	0.004
	Antieczematic	0.771	0.025

^†^ Probable activity (Pa) and inactivity (Pi) thresholds: Only predictions with Pa > Pi were considered. Pa ≥ 0.7 represents a high probability of observable biological activity.

## Data Availability

The data supporting the reported results are available at https://data.mendeley.com/drafts/7xvd9pmwc9 (accessed on 27 May 2025).

## Supplementary Materials

The following supporting information can be downloaded at: https://www.mdpi.com/article/10.3390/antiox15030291/s1, Table S1: In silico prediction toxicity properties.
