# Supplementary material for: Comprehensive Characterization of Lantana camara Essential Oil from Angola: GC-MS Profiling, Antioxidant Capacity, and Drug-likeness Prediction
Source: Antioxidants (Basel). 2026 Feb 26;15(3):291. doi: 10.3390/antiox15030291 (PMC13024179; doi:10.3390/antiox15030291)
Supplement: Supplementary file 1 [file antioxidants-15-00291-s001.zip › antioxidants-4108282-supplementary.pdf]

Supplementary Material

***Table S1: In silico prediction toxicity properties***

Comprehensive ADMET profiling was conducted using a multi-platform approach. In addition to ADMETlab and PASS, the results were cross-referenced with Osiris, Deep-PK, pkCSM, and preADMET to ensure predictive consistency, with findings presented below:

| N° | **Compound** | **Toxicity risks** |
|---|---|---|
| 1 | α-humulene | A[1,2,4],D[4],E[4],F[2] |
| 2 | sabinene | A[1,2,4],D[2,4],E[4],F[1,2,4],H[2] |
| 3 | bicyclogermacrene | A[1-4],B[3],C[3],D[4],E[4],F[1,2,4] |
| 4 | β-caryophyllene | A[1,2,4],D[4],E[4],F[1,2,4] |
| 5 | 1,8-cineole (Eucalyptol) | A[2-4],B[3,6],F[2,4,5],G[3] |
| 6 | nerolidol | A[1,2,4],E[1],F[2,4,5],H[2] |
| Irritant (A), mutagenic (B), tumorigenic (C), carcinogenicity (D), hepatotoxicity (E), skin sensitization (F), reproductive effects (G) , respiratory effects (H); data obtained by PASS[1], ADMETlab3.0[2], Osiris[3], Deep-PK[4], PkCSM[5], preADMET[6] | | |
